# Cutaneous Rosai-Dorfman Disease: A Case Report

**DOI:** 10.7759/cureus.39617

**Published:** 2023-05-28

**Authors:** Kayla St. Claire, Manar Edriss, Geoffrey A Potts

**Affiliations:** 1 Dermatology, Wayne State University School of Medicine, Detroit, USA; 2 Internal Medicine, St. Joseph Mercy Ann Arbor Hospital, Ypsilanti, USA

**Keywords:** skin nodule, rosai dorafman disease, non-langerhan, histiocytosis, cutaneous

## Abstract

Rosai-Dorfman disease (RDD) is a rare benign non-Langerhans cell histiocytosis. The most common site of extranodal involvement is the skin. Cutaneous involvement without lymphadenopathy is extremely rare. It is often difficult to diagnose primary cutaneous RDD secondary to the non-specific nature of its clinical and histologic features. Consequently, diagnosis can be significantly delayed. To our knowledge, about 220 reports of purely cutaneous RDD are documented in the literature to date. We present an additional unique case of cutaneous RDD and emphasize the challenging nature of accurate clinical and histopathologic diagnosis.

## Introduction

Rosai-Dorfman disease (RDD), also known as sinus histiocytosis with massive lymphadenopathy, is a rare benign non-Langerhans cell histiocytosis. Rosai and Dorfman first described it as a clinicopathologic entity in 1969 [1]. The etiology behind RDD remains unknown, but due to its sporadic nature, viral and autoimmune causes have been hypothesized. RDD may be limited to the lymph nodes or have extranodal involvement, which occurs in more than 40% of patients [2]. The most common site of extranodal involvement is the skin [1]. Cutaneous involvement without lymphadenopathy is extremely rare. To our knowledge, documentation in the literature is currently limited, with only about 220 described cases of purely cutaneous RDD available. We present an additional unique case of cutaneous RDD and emphasize the challenging nature of accurate histopathologic diagnosis.

## Case presentation

A 65-year-old African American male presented to the clinic with asymptomatic nodules on the right lower abdomen. It initially started three years prior as a small papule that enlarged and resolved spontaneously. It recurred within the past year but persisted, increased in number, and enlarged to its present size. He denied bleeding, draining, or preceding trauma. Review of systems was negative for fevers, chills, unintended weight loss, night sweats, lymphadenopathy, gastrointestinal symptoms, or similar lesions elsewhere. Past medical history was significant for type 2 diabetes mellitus, hypertension, and atrial fibrillation. He denied a history of cancer. He denied a family history of any skin conditions or similar lesions. 

**Figure 1:**
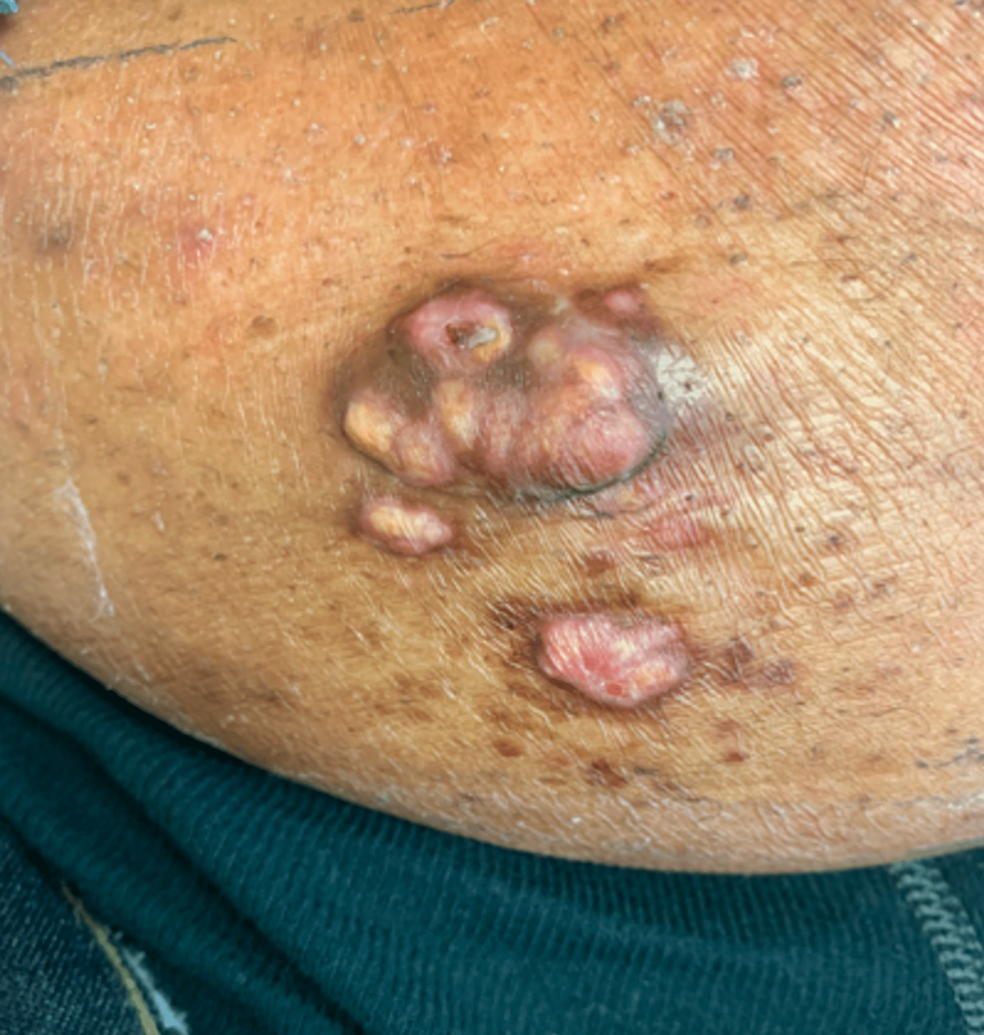
Pink to yellow firm, indurated, nodules coalescing into a 3 cm tumor with two similar appearing nodules of smaller size located inferiorly on the right lower abdomen.

Physical exam of the right lower abdomen revealed pink to yellow firm, indurated, nodules coalescing into a 3 cm tumor with two similar appearing nodules of smaller size located inferiorly (Figure 1). All lesions had a perimeter of hyperpigmentation. The lesions were non-tender to palpation. There was no cervical, axillary, or inguinal lymphadenopathy. Comprehensive metabolic panel was only remarkable for elevated glucose of 123. Hemoglobin A1c was elevated at 6.7. The lipid profile and thyroid stimulating hormone were within normal limits. Findings were concerning for several differentials including but not limited to RDD, Langerhans cell histiocytosis, sarcoidosis, acneiform eruptions, cutaneous lymphoma, Kaposi sarcoma, granuloma annulare, eruptive xanthoma, dermatofibrosarcoma protuberans, and leukemia cutis. 

**Figure 2:**
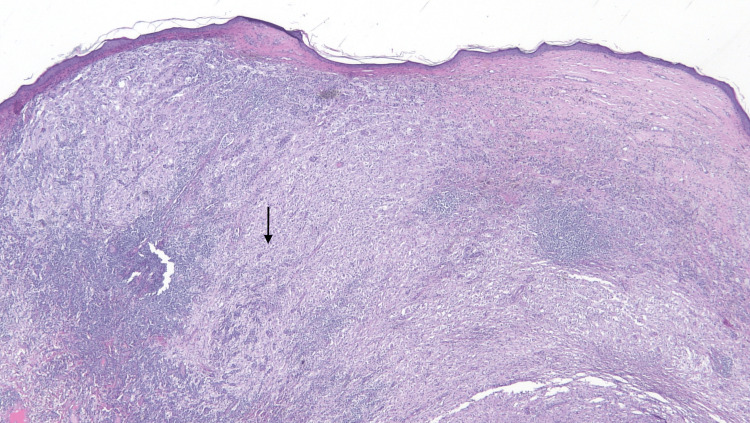
A nodular proliferation of non-lymphoid histiocytic infiltrate consisting of eosinophilic-appearing histiocytes containing multiple nucleoli on H&E stain with magnification x20. H&E: Hematoxylin and eosin

**Figure 3:**
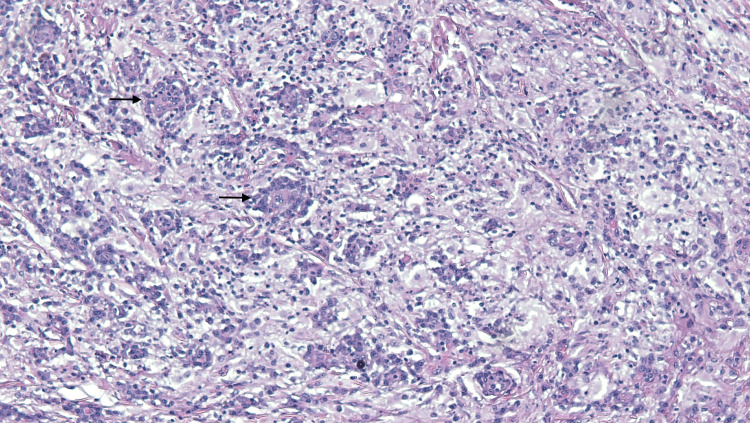
Foamy histiocytes surrounded by small lymphocytes with numerous areas of emperipolesis on H&E stain with magnification x100. H&E: Hematoxylin and eosin

**Figure 4:**
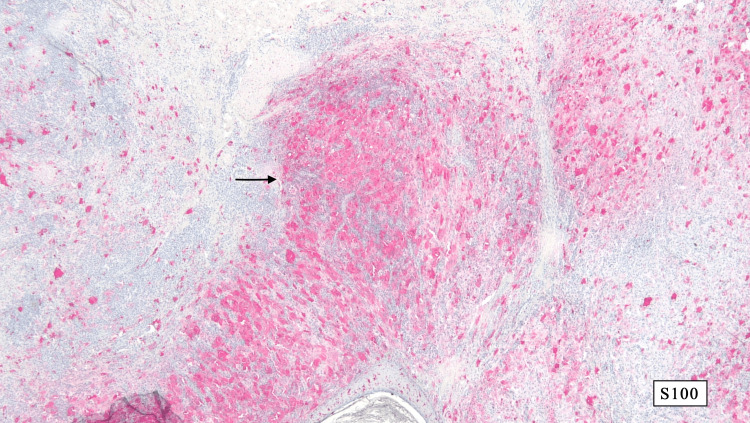
The histiocytic infiltrate demonstrated positivity for S-100 with magnification x20.

A 4mm punch biopsy of the right lower abdomen demonstrated acutely inflamed granulation tissue and proliferation of capillary-like blood vessels with some vascular channels and cystic spaces lined by foamy histiocytes with plump nuclei. A lymphoplasmacytic infiltrate extended to the deep dermis. CD-31 showed excessive background staining. CD-34 highlighted a few small capillary-like blood vessels. CD-163 highlighted numerous histiocytes. SOX-10 and pan-cytokeratin were negative. Four months later, an excisional biopsy was performed for definitive diagnosis. It showed a nodular proliferation of non-lymphoid histiocytic infiltrate consisting of eosinophilic appearing histiocytes containing multiple nucleoli (Figure 2). The foamy histiocytes were surrounded by small lymphocytes with numerous areas of emperipolesis (Figure 3). Histiocytic infiltrate was positive for S-100 (Figure 4) and CD163 while negative for CD1a, Melan-A, pan-cytokeratin, and SOX-10. These histochemical findings support the diagnosis of RDD.

The patient was instructed to obtain a complete blood count with differential and computed tomography of the abdomen/pelvis with IV contrast to rule out systemic associations and lymphadenopathy. No adenopathy was seen on imaging. He was offered excision of remaining nodules.

## Discussion

Compared to patients with systemic RDD, cutaneous RDD presents at an older mean age of about 43.5 years, is more common in White and Asian females, and has no lab abnormalities. Cutaneous manifestations are non-specific with asymptomatic red-brown to yellow papules, nodules, or plaques of varying size that can be confined or disseminated, without predisposition for any distinct anatomic site. The most common sites of involvement are thought to be the extremities [3]. The diagnosis of primary cutaneous RDD is often difficult and significantly delayed due to the non-specific nature of its clinical and histologic features [4]. Even after multiple biopsies, clinicians may struggle to make the diagnosis. The histologic qualities of primary cutaneous disease are often only recognized as non-specific inflammation. We present a unique case of cutaneous RDD as our patient was over 20 years older than the mean age of diagnosis, African American, and male; all factors that are less typically seen in this condition. We also emphasize the challenging nature of accurate clinical diagnosis in that its presentation leads to a broad differential, as well as challenging histopathologic diagnosis, in that it may require several/large biopsies to identify the well-recognized features of RDD.

A large series of cutaneous RDD was described by Kong et al. in their clinical and histopathologic study of 25 patients in China [5]. Of the 39 skin lesions that were observed, the extremities were noted to be the most commonly involved, followed by the trunk and the face. The most common clinical presentation was the papulonodular type (79.5%), followed by the indurated plaque type (12.8%) and tumor type (7.7%). Unique histologic cases in this cohort were the identification of fibrosis in four patients, xanthomatous change in two cases, and coexistence of localized Langerhans cell histiocytosis in one patient. Follow-up was observed in 22 patients over 2-55 months where they found that surgical excision was the most exclusive effective treatment. Additionally, Ahmed et al. performed a review of the 220 patients with purely cutaneous RDD published to date and found the majority of cases presented with multiple lesions (60%) that remain present for an average timespan of 19 months [3]. Eleven percent of these cases were associated with IgG and 14.5% with increased erythrocyte sedimentation rate. Other rare but associated findings included uveitis, Crohn’s disease, positive EBV, HHV, HIV, HSV, CMV, and CRD that arise in VZV scars, concurrent LCH in the same specimen, and concurrent marginal zone lymphoma [3].

Histopathology of cutaneous RDD typically shows a dermal infiltrate of large polygonal histiocytes with abundant pale to eosinophilic cytoplasm admixed with an inflammatory infiltrate [6]. Emperipolesis, which is the presence of intact inflammatory cells like lymphocytes, plasma cells, and neutrophils within the cytoplasm of histiocytes, is characteristic of the disease. The histiocytes stain positively for S100 and CD68 but are negative for CD1a [4]. Superficial and small biopsies should be avoided to aid in diagnostic accuracy. This was evidenced in our case in which 4 mm punch biopsy was non-specific and ultimately excisional biopsy led to definitive diagnosis. Diagnostic histologic challenges may include increased foamy cells effacing the epidermis, vascularity, stromal reaction, inflammation leading to ulceration which may be mistaken for xanthomatous lesions, hemangiomas/vasculitis, fibrous soft-tissue tumors, or malignant processes, respectively.

The recommended management of RDD is close observation, as it is usually an indolent, benign self-limited condition. If treatment is desired, surgical excision is considered most effective for both solitary disease and multifocal disease. Improvement has been observed with topical, intralesional, or systemic corticosteroids, cryotherapy, radiation, retinoids, methotrexate, high-dose thalidomide, vincristine, dapsone, and imatinib with mixed results. 

## Conclusions

Diagnosing cutaneous RDD is challenging due to the variations in its clinical and histologic presentation. Our case is unique as this patient was over 20 years older than the mean age of diagnosis, African American, and male, all factors that are less typically seen in this condition. This case also emphasizes the challenging nature of accurate clinical and histopathologic diagnosis. Key features of histopathology include emperipolesis and dermal infiltrate of histiocytes that stain positively for S100 and CD68 and negatively for CD1a. Management involves observation although surgical excision, and several treatment options are available if therapy is desired.
